# Parathyroid hormone promotes maxillary expansion and reduces relapse in the repeated activation maxillary expansion rat model by regulating Wnt/β-catenin pathway

**DOI:** 10.1186/s40510-021-00394-0

**Published:** 2022-01-03

**Authors:** Mengting Xu, Yuan Li, Xiaoxia Feng, Wei Zheng, Zhihe Zhao, Yu Li

**Affiliations:** 1 State Key Laboratory of Oral Diseases, National Clinical Research Center for Oral Diseases, Department of Orthodontics, West China Hospital of Stomatology, Sichuan University, #14, 3rd Section, South Renmin Road, Chengdu, 610041, People’s Republic of China; 2 The Affiliated Stomatology Hospital, Key Laboratory of Oral Biomedical Research of Zhejiang Province, Zhejiang University School of Medicine, Hangzhou, People’s Republic of China; 3 State Key Laboratory of Oral Diseases, National Clinical Research Center for Oral Diseases, Department of Oral and Maxillofacial Surgery, West China Hospital of Stomatology, Sichuan University, #14, 3rd Section, South Renmin Road, Chengdu, 610041 People’s Republic of China

**Keywords:** Parathyroid hormone, Bone remodeling, Maxillary expansion, Mid-palatal suture, Relapse

## Abstract

**Background:**

Constricted maxillary bone is a common skeletal deformity, which may lead to crowding and posterior crossbite. Mid-palatal suture expansion is often used to increase the maxillary width, but its skeletal effects are limited and tend to relapse, even with prolonged retention. We hypothesized that parathyroid hormone (PTH) may reduce the relapse of maxillary expansion.

**Methods:**

We established a novel rat maxillary expansion model using palatal tubes with an insertable “W”-shaped spring which can be repeatedly activated. A total of 32 male healthy Wistar rats were randomly divided into six groups: the control group, the PTH group, the expansion group, the expansion + PTH group, the expansion + relapse group and the expansion + PTH + relapse group. All animals in the first 4 groups were killed after 10 days and the 2 relapse groups were killed after 15 days. The maxillary arch widths and histological staining were used to assess the expansion and relapse effects. The immunohistochemical staining, micro-CT, RT-qPCR and Western blot were used to evaluate the bone remodeling during expansion.

**Results:**

The suture width was increased by the expansion device, and the repeated activation maxillary expansion rat model showed better expansion effects than the conventional model. PTH significantly promoted the expansion width and reduced the relapse ratio. Meanwhile, in the expansion + PTH group, histological and immunohistochemical staining showed that osteoblasts, osteoclasts, new cartilage and osteoid were significantly increased, micro-CT showed increased bone mass, and PCR and Western blot results confirmed up-regulation of RANKL, β-catenin, type II collagen and OCN.

**Conclusion:**

The novel repeated activation maxillary expansion rat model has better effects than the conventional model. PTH enhances the maxillary expansion and reduces its relapse by regulating Wnt/β-catenin and RANKL pathways. PTH administration may serve as an adjunctive therapy in addition to mechanical expansion for treatment of maxillary constriction.

## Background

Maxillary constriction is one of the most common craniofacial deformities, which can lead to crowding, posterior crossbite, anterior open bite and other types of malocclusion [1]. Its etiology is complex, generally believed to be associated with bad habits such as finger sucking or oral breathing [2]. Nowadays, maxillary expansion is a commonly used treatment approach to expand the mid-palatal suture and increase the maxillary width [3]. The effects of expansion include mid-palatal suture expansion, tooth displacement, tooth inclination and alveolar process inclination, among which mid-palatal suture expansion is the real skeletal effect [4]. However, the skeletal expansion effect achieved by mid-palatal suture expansion only accounts for 15–50% of the total increase in arch width [5], while relapse may also occur even after retention because of the insufficient bone remodeling [6, 7]. Therefore, increasing expansion width and reducing relapse through improving bone remodeling during mid-palatal suture expansion play a key role in the improvement in maxillary constriction treatment.

Previous researches have shown that in the physiological state, the reconstruction of palatal suture tissue is a process of bone resorption and bone formation simultaneously under the expansion force [8]. Osteoclast activation promotes bone resorption, and mesenchymal precursors promote the formation of bone and cartilage. When subjected to expansive force, both intramembranous and endochondral osteogenesis occur in suture expansion [9], and the former is considered more critical according to some studies [10, 11]. Based on the complex bone remodeling and modeling processes in mid-palatal suture expansion, various interventions have been attempted in recent years to enhance its widening effect, such as growth factors (TGF-1 [12], Nell-1, BMP [13]), drugs (bisphosphonates [14], thymoquinone [15]), physical stimulation (laser [16, 17], enhancement frequency [18]), and alternate expansion and constriction [19].

Parathyroid hormone (PTH) is secreted by chief cells of parathyroid gland as an important regulator of calcium and phosphorus metabolism [20]. PTH was reported to promote bone remodeling, and teriparatide (PTH1-34) was approved by FDA as an anabolic therapy for clinical treatment of osteoporosis [21]. Studies have shown that PTH has bidirectional regulating effects on bone remodeling and promotes bone resorption during continuous administration [21–23]. For example, chronic overdose of PTH caused by primary hyperparathyroidism or secondary calcium deficiency could increase the rate of bone remodeling and lead to bone loss, while interval administration of PTH could promote bone formation. Daily injection of PTH is the only anabolic treatment for patients with osteoporosis at high risk of fracture. During the intermittent injection of PTH, osteoblasts and bone formation increased rapidly because of the increased rate of bone remodeling, which leads to more bone formation than bone absorption. At the same time, it can increase bone formation by promoting the proliferation of osteoblast precursors and inhibiting the apoptosis of osteoblasts. Recent studies have also suggested that PTH facilitated bone resorption by binding to the PTHR1 receptor of osteoblast and promoted osteoclast formation and activation by up-regulation of RANKL expression. In addition, studies have confirmed the effects of PTH in orthodontic tooth movement [24], healing of alveolar fossa [25] and restoration of maxillary defect [26].

Thus, we hypothesize that PTH may play a role in the bone remodeling process of mid-palatal suture expansion through promoting both osteoclastogenesis and osteogenesis. In the present study, we established a novel repeated activation maxillary expansion rat model and further explored the therapeutic effects and possible mechanisms of PTH in combination with maxillary expansion to increase the skeletal maxillary expansion and reduce relapse.

## Materials and methods

### Establishment of the repeated maxillary expansion rat model

**Fig. 1:**
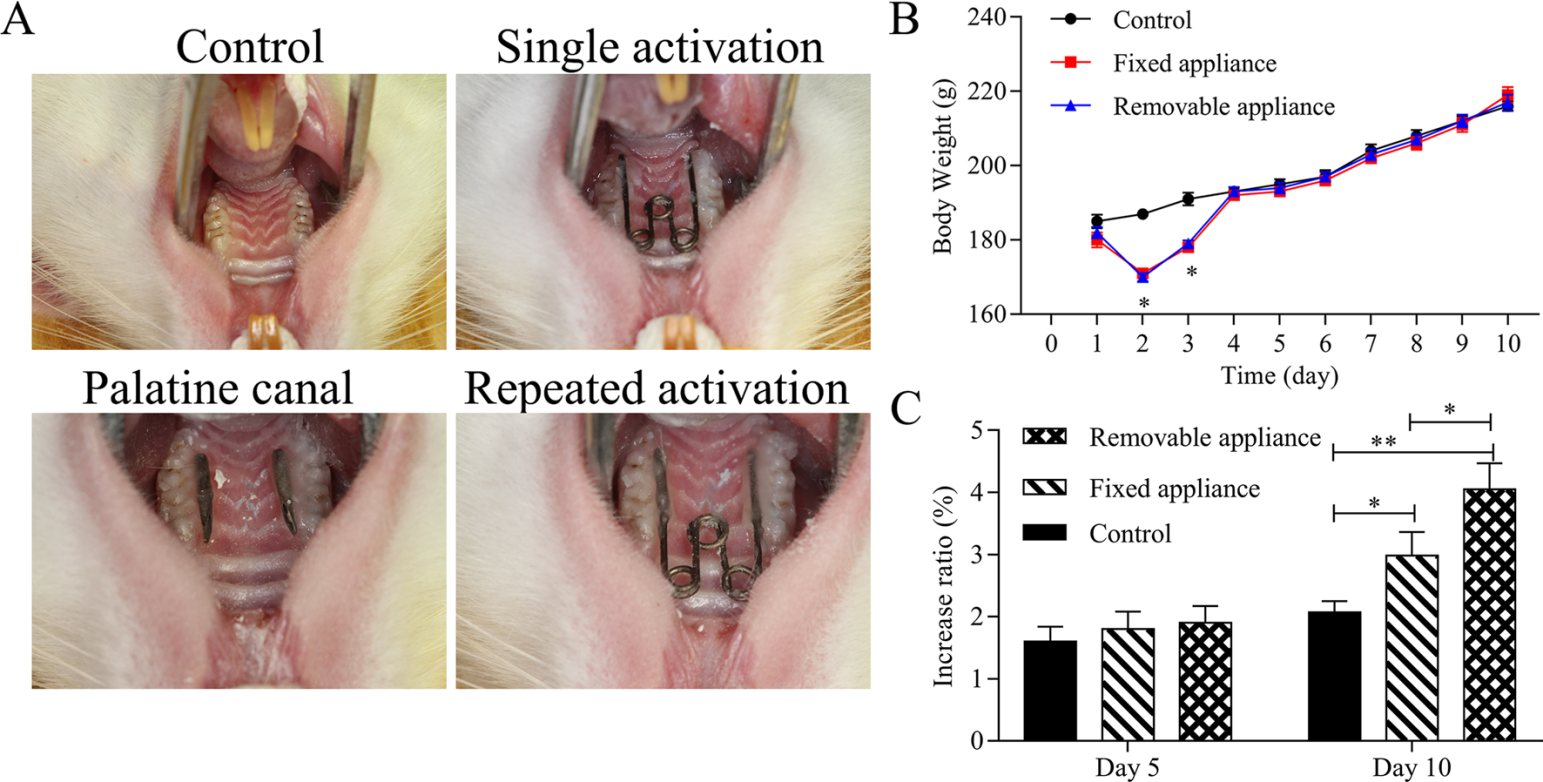
The changes of body weights and maxillary arch widths of maxillary expansion model rats. **A** Establishment of maxillary expansion model rats with continuous force. **B** The body weights of rats were measured after maxillary expansion. **C** The maxillary arch widths of rats were examined after maxillary expansion, and the increase ratio was counted. *P < 0.05, **P < 0.01 versus control group

A total of 24 male healthy Wistar rats (6 weeks old, mean body weight 180 g) were provided by the Animal Experimental Center of Sichuan University. The feeding conditions mainly included available food and water, 50–60% humidity, 22 ± 2 °C and 12 h lights every day. All the animal protocols were approved by the ethics committee of State Key Laboratory of Oral Diseases of Sichuan University (SKLODLL2013A163). After a 7-day acclimation to the laboratory environment, the rats were randomly divided into three groups (*n* = 8 in each): the control group, the single activation (SA) group and the repeated activation (RA) group. In each group, the rats were intraperitoneally injected with 10% chloral hydrate solution (0.35 ml/100 g) and exposed the hard palate tissue and maxillary molars. We adopted conventional expansion device of single activation for the SA group and the novel expansion device of repeated activation for the RA group. The conventional expansion device is a “W”-shaped spring made of a 0.014-inch Australian stainless steel wire (the length of free arms on both sides is 10 mm), which was bonded to the palatal sides of the maxillary molars. The novel repeated expansion device involves palatal tubes bonded on the palatal sides of the maxillary molars, and a “W”-shaped spring which is inserted into the palatal tubes and can be taken out for reactivation (Fig. 1A). When the spring is compressed from the initial width to the inner width of the mouth, force of about 40 g is generated. The rats were checked daily, and once the device dropped, it was rebonded immediately. Two days after the operation, one of the expansion devices in the SA group was loose and was immediately re-glued, while the rest did not fall off until the animals were killed. Weight changes were recorded daily. Before expansion and after expansion for 5 and 10 days, the maxillary arch width was measured with a vernier caliper between the mesial buccal neck of the second molars, which is approximate to the width of the maxillary alveolar crest. All the rats were killed after expansion for 10 days.

### PTH administration

The PTH powder was centrifuged, and sterile PBS solution was added to make 20% PTH solution. A total of 32 male healthy Wistar rats (6 weeks old, mean body weight 180 g) were randomly divided into four groups (*n* = 8 in each): the PTH group which was subcutaneously injected with PTH solution (4 μg/100 g body weight) daily, the expansion (E) group which wear the RA expansion device and was subcutaneously injected with the same amount of PBS daily, the expansion + PTH (E + PTH) group which both wore the RA expansion device and was subcutaneously injected with PTH solution (4 μg/100 g body weight) daily, and the control (C) group which was subcutaneously injected with the same amount of PBS daily. The initial force of expansion was 40 g in the E and E + PTH groups, and the device was taken out and reactivated after 5 days to maintain the force. Weight changes were recorded daily, and the maxillary arch width was measured with a vernier caliper at the 0, 5 and 10 days. All the rats of the C and PTH groups were killed at the 10 days, while in each of the E and E + PTH groups 5 rats were randomly sacrificed at the 10 days, and the expansion devices were removed from the remaining 3 rats which were sacrificed after 5 days of natural retention to observe the relapse, called the expansion + relapse (E + R) group and the expansion + PTH + relapse (E + PTH + R) group. In the course of this experiment, no device fell off.

### Micro-computed tomography (μCT) analysis

Animals were killed by excessive chloral hydrate after 10 days, and the samples were washed with PBS, fixed with 10% formalin for 24 h. Micro-computerized tomography (Inveon Micro-PET/CT, Siemens) was used to scan the specimens (scanning software was Inveon Acquisition Workplace, scanning voxel size was 15 μm, X-ray tube voltage was 70 kV, and current was 200 μA). The region of interest (ROI) was defined as a cuboid (10 mm * 2 mm * 2 mm) whose center was the intersection of the longitudinal plane at the mesial buccal tips of bilateral second molars and the mid-palatal suture (Fig. 5A). The images were segmented, registered and quantified, and the spatial parameters were analyzed, including trabecular bone volume fraction (bone volume/total volume, BV/TV), smaller specific bone surface (BS/BV), trabecular number (TbN), trabecular thickness (Tb.Th) and trabecular separation (Tb.Sp).

### Histochemical staining

After the micro-computed tomography scan, the samples were decalcified with 10% EDTA solution for 2 months, dehydrated with gradient ethanol and treated with dimethylbenzene. Then, the samples were sectioned to obtain 7-μm-thick continuous tissue sections from the maxillary coronal plane. Then, 7-μm sections were stained with H&E solution (H8070, Solarbio, China). For Masson staining, the sections were treated with Ponceau solution for 5 min, phosphomolybdic acid solution for 4 min and aniline blue solution for 10 min. For toluidine blue staining, the sections were dyed with toluidine blue solution for 2 min. After washing for three times with distilled water, the sections were treated with 95% ethanol, dehydrated, transparent and sequestered. Finally, the images were observed using a microscope (OLYMPUS CX41). Each type of cells was analyzed and quantified by using an image analysis system (Image-Pro; Media Cybernetics, Silver Spring, MD, USA) according to the previous study [27].

### TRAP staining

Firstly, the incubation buffer was obtained using 8 ml acetic acid buffer (0.2 mol/l, XY-299-67811), 1 ml O-(7-Azabenzotriazol-1-yl)-N,N,N',N'-tetramethyluroniumhexafluorophosphate (P311024) and 1 ml naphthol AS-BI phosphate (20 mg/ml, Mitachieve, SAG606682). Adjust pH to 5.0, 141 mg potassium sodium tartrate (HPBIO226010) was added to the solution. After washing with double distilled water, the sections were incubated with the incubation buffer for 1 h, treated with hematoxylin staining solution (YB130822-15) for 2 min, and were washed with alkali solution. After sealing, the results were observed using a microscope (OLYMPUS CX41).

### Immunohistochemical (IHC) assay

The sections were treated with 3% H_2_O_2_ for 25 min at room temperature in dark and washed with PBS for three times. Then, the slides were incubated with 3% BSA at room temperature for 30 min and primary antibody (Collagen type I, Biorbyt, orb420618) at 4 °C overnight. And then the slides were incubated with secondary antibodies at room temperature for 50 min and 3,3'-diaminobenzidine (DAB). Finally, slides were stained with hematoxylin and dehydrated with different concentrations of alcohol. The results were obtained using a microscope (OLYMPUS CX41).

### Quantitative real-time PCR (RT-qPCR) assay

Fresh specimens of maxillary palatal suture with a width of 2 mm were intercepted and quickly ground in liquid nitrogen, and then total RNAs were prepared from the grinding fluid using Trizol reagent (Invitrogen) in line with the instructions. According to the manufacturers’ instructions, cDNA was produced using the RevertAid™ First Strand cDNA Synthesis Kit (5201) based on extracted RNAs. The expressions of RANKL, β-catenin, collagen II and OCN were quantified using BestarTM qPCR Master Mix (DBI Bioscience) on an ABI 7500 detection System (Applied Biosystems). Glyceraldehyde 3-phosphate dehydrogenase (GAPDH) was used as an internal control. According to the Ct value, the data were analyzed using 2^−△△Ct^ calculation. The sequences of primers were GAPDH primers: 5'-GGATGCAGGGATGATGTTCT-3' (forward primer) and 5'-AACTTTGCCATTGTGGAAGG-3' (reverse primer); RANKL primers: 5'-TGATTCATGTAGGAGAATTAAACAGG-3' (forward primer) and 5'-GATGTGCTGTGATCCAACGA-3' (reverse primer); β-catenin primers: 5'-TCCCAGTCCTTCACGCAAGAG-3' (forward primer) and 5'-GTGGCAAGTTCCGCGTCATC-3' (reverse primer); collagen II primers: 5'-CATCACCTACCACTGCAAGAAC-3' (forward primer) and 5'-ACGTCGAAGCCGAATTCC-3' (reverse primer); OCN primers: 5'-GTGCAGCCTTTGTGTCCAAG-3' (forward primer) and 5'-GTCAGCCAACTCGTCACAGT-3' (reverse primer).

### Western blot assay

Fresh specimens of maxillary palatal suture were quickly ground in liquid nitrogen, and the total proteins were extracted from the grinding fluid using the RIPA buffer supplementing phenylmethylsulfonyl fluoride (PMSF). Based on the manufacturers’ instructions, the BCA kit (Pierce, Rockford) was utilized to evaluate the concentrations of proteins. Proteins (40 μg) in each group were isolated by 10% SDS-PAGE and then transferred onto nitrocellulose membranes (Millipore, Billerica, MA, USA). After treatment with 5% low fat milk for 2 h, the membranes were incubated with primary antibodies including anti-RANKL (Abcam, ab9957), anti-collagen II (Abcam, ab34712), anti-OCN (Abcam, ab13420), anti-β-catenin (Cell signaling, #9562) and anti-β-actin (Abcam, ab8227) at 4 °C overnight. Next day, the membranes were incubated with horseradish peroxidase-conjugated Donkey Anti-Rabbit secondary antibodies at room temperature for 2 h. The results were observed and obtained by the enhanced chemiluminescent (ECL) reagents (EMD Millipore) according to the manufacturers’ instructions.

### Statistical analysis

SPSS (version 19.0, SPSS Inc., Chicago, USA) was used to process data analysis. All experiments were repeated three times, and the data were all expressed as Mean ± SD. The difference between groups was analyzed by one-way ANOVA. *P* value less than 0.05 was considered statistically significant.

## Results

### The RA model expanded the maxillary arch and promoted bone remodeling more efficiently than the SA model

The body weights of rats were significantly decreased in the single activation (SA) group and the repeated activation (RA) group compared with the control group on day 2 and 3 (*P* < 0.05), while the body weights had no difference in each group after 3 days (Fig. 1B). The increased maxillary arch width of the RA group was significantly larger than that of the SA or control group on day 10 (*P* < 0.05, Fig. 1C).

**Fig. 2:**
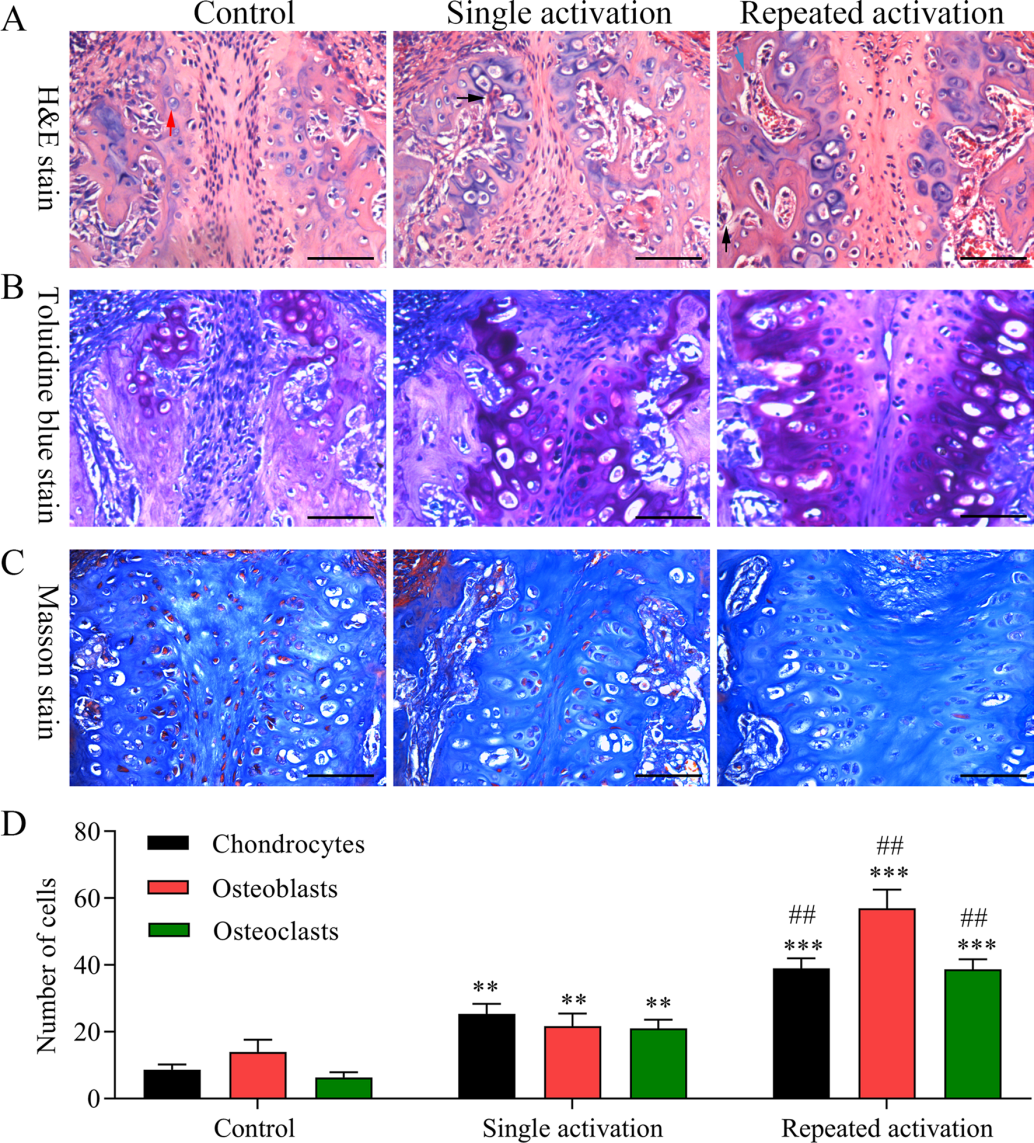
The effects of maxillary expansion on bone remodeling in rats. The rats were divided into control, single activation and repeated activation groups, respectively. **A** The conditions of palatal suture tissue remodeling and widening were assessed by H&E staining. Magnification, 400 ×. **B** The chondrocytes in mid-palatal suture were evaluated by toluidine blue staining. Magnification, 400 ×. **C** The histological fibrosis grades of mid-palatal suture in rats were measured by Masson staining. Magnification, 400 ×. **D** The number change of chondrocytes, osteoblasts and osteoclasts was counted in each group based on the H&E staining results. **P < 0.01, ***P < 0.001 versus control group; ##*P* < 0.01 versus single activation group. The red arrow indicates chondrocyte, the black arrow indicates osteoclast, and the blue arrow indicates osteoblast

In order to further explore the effects of maxillary expansion on bone remodeling, H&E staining, toluidine blue staining and Masson staining were performed. As the H&E staining results showed, there was no obvious tissue necrosis in each group. The mid-palatal suture in the control group showed that mesenchymal precursors were located in the middle, and mature chondrocytes were situated on both sides. A few osteoblasts could also be seen at the junction of bone plate and cartilages. The mid-palatal suture in the SA group indicated that the mid-palatal suture was dilated, mesenchymal precursors proliferated, cartilages increased, and there were osteoclasts and bone resorption area at the periosteal margins. The mid-palatal suture in the RA group revealed that the mid-palatal suture tissue was prominently widened, while cartilages, osteoblasts and osteoclasts were observably increased (Fig. 2A, D). The results from toluidine blue staining indicated that chondrocytes were markedly increased in the SA or RA group rather than the control group, and the increase was most pronounced in the RA group (Fig. 2B). The results from Masson staining showed that in the control group, the fibers in mid-palatal suture arranged regularly; in the SA group, the fibers crisscrossed and twisted; in the RA group, the fibers were obviously seen through the enlarged middle palatal suture (Fig. 2C). Therefore, we suggested that our novel maxillary expansion model could expand the maxillary arch and improve bone remodeling more efficiently than the conventional rat model.

### PTH increased maxillary arch widths, promoted bone remodeling and reduced relapse in the rat maxillary expansion model

**Fig. 3:**
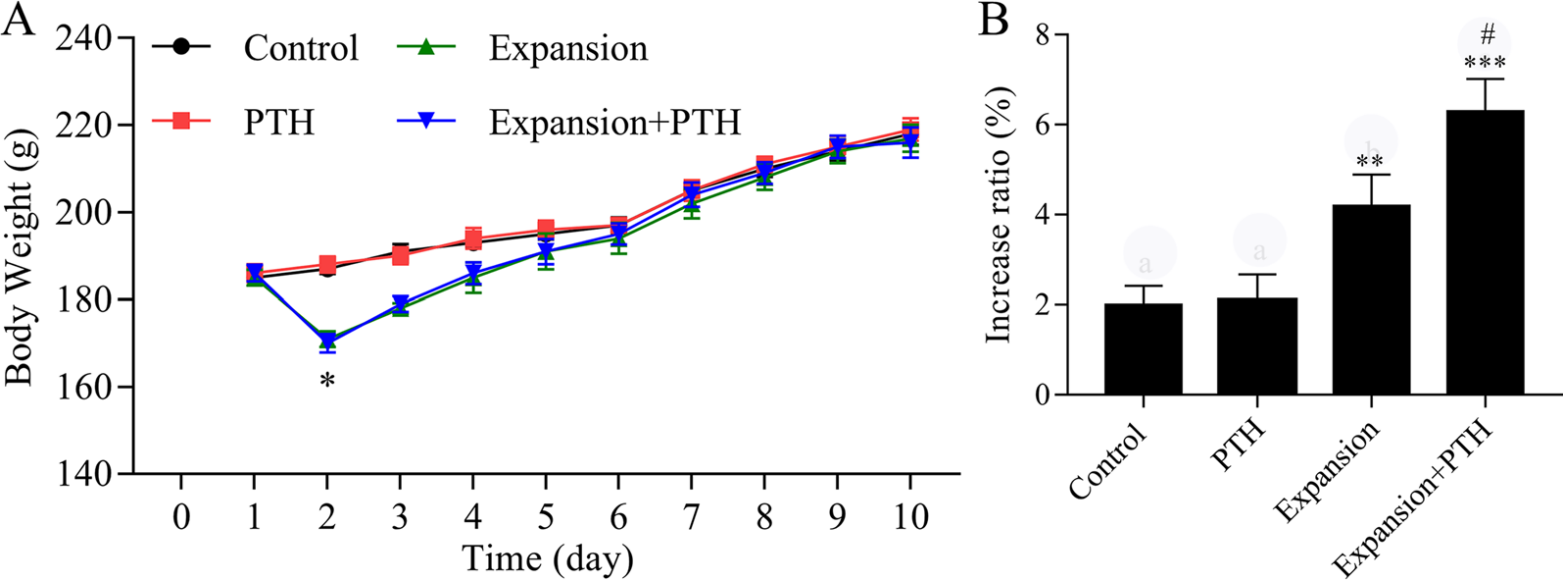
PTH increased maxillary arch widths of maxillary expansion model rats. The rats were divided into control, PTH, expansion and expansion + PTH groups, respectively. **A** The body weights of rats were detected in each group, *P < 0.05 versus control group. **B** The maxillary arch widths of rats were measured in each group. *P < 0.05 versus control group; #*P* < 0.05 versus expansion group

In order to further investigate the effects of PTH on maxillary expansion, PTH was used to treat the rats in the control and the RA expansion groups. The results showed that the body weights of rats were gradually increased over time, and the body weights were decreased in the expansion and expansion + PTH groups compared with the control and PTH groups on day 2 after surgery, respectively, and then recovered on day 3 (*P* < 0.05, Fig. 3A). The maxillary arch widths were significantly increased in the E and E + PTH groups; compared with the E group, the maxillary arch widths were significantly increased in the E + PTH group (*P* < 0.05, Fig. 3B). We also found that two rats had relapses (the relapsed ratio is 66.7%) in the expansion group, and no rat had relapse (the relapsed ratio is 0%) in the expansion + PTH group.

**Fig. 4:**
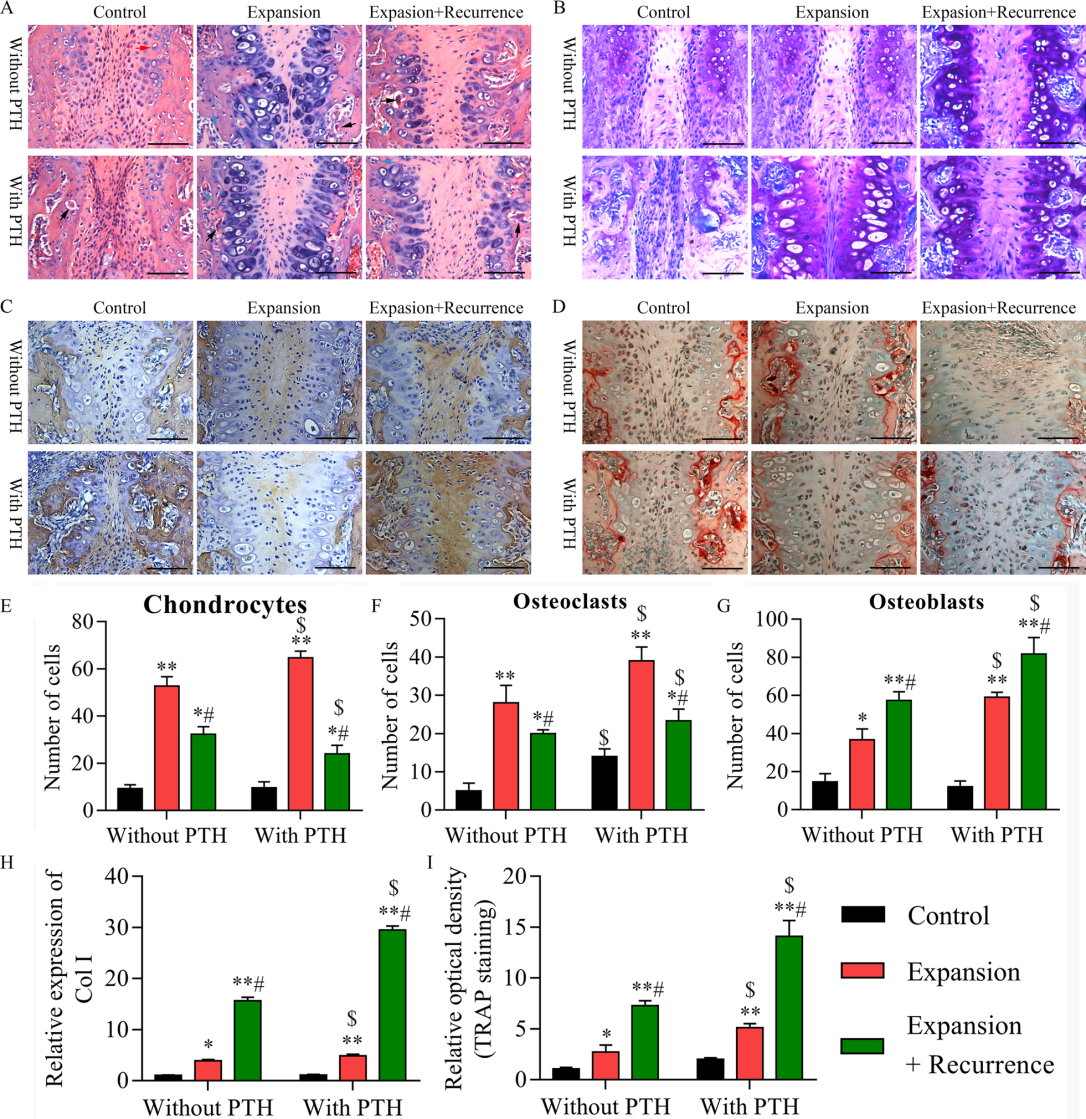
PTH promoted bone remodeling and reduced relapse in the process of maxillary expansion. The rats were divided into control, PTH, expansion, expansion + PTH, expansion + relapse and expansion + relapse + PTH groups, respectively. **A** H&E staining was performed to evaluate the conditions of palatal suture tissue remodeling and widening. Magnification, 400 ×. **B** Toluidine blue staining was utilized to assess the changes of chondrocytes in each group. Magnification, 400 ×. **C** IHC assay was used to detect the new bone formation in each group. Magnification, 400 ×. **D** TRAP staining was used to assess the osteoclasts and bone resorption in each group. Magnification, 400 ×. The white arrow indicates osteoclast ,and the blue arrow indicates regenerated new bone. Based on the H&E staining results, the quantitative analysis of **E** chondrocytes, **F** osteoclasts, **G** osteoblasts was calculated in control, PTH, expansion, expansion + PTH, expansion + relapse and expansion + relapse + PTH groups. **H** The expression of collagen type I was counted based on the IHC results. **I** The relative optical density was counted according to the TRAP staining results. *P < 0.05, **P < 0.01 versus control group; #*P* < 0.05 versus expansion group; $*P* < 0.05 versus without PTH group. The red arrow indicates chondrocyte, the black arrow indicates osteoclast, and the blue arrow indicates osteoblast

Our results of H&E staining indicated that no obvious tissue necrosis was observed in all specimens; the mid-palatal suture tissues expressed normal physiological structure in the control group; in the PTH group, the mid-palatal suture had no significant structural change, and a small number of osteoclasts were present at the margin of the bone; in the E group, mesenchymal precursors proliferated, cartilages increased, and osteoblasts and osteoclasts were present at the periosteal margins; in the E + PTH group, osteoid, cartilage, osteoclast and osteoblast were obviously increased; in the E + R group, chondrocytes began to degenerate and new bone was formed in the suture of the palate; the E + PTH group had more chondrogenesis than the E group; in the E + PTH + R group, after the removal of the maxillary expansion device, more chondrocytes differentiate into bone under buccal compressive pressure, which could protect against relapse by increasing bone mass (Fig. 4A, E–G). The results of toluidine blue staining showed that the distribution of mid-palatal suture cartilage in PTH group was similar to that in the control group; in the E group, the range of chondrocytes expanded; in the E + PTH group, the cartilage range was increased, and more immature cartilage stroma was observed; in the E + R group, chondrocytes became enlarged and degenerated; in the E + R + PTH group, the cartilage degenerated, which was replaced by new bone (Fig. 4B). The results from Collagen type I staining revealed that mesenchymal progenitor cells of mid-palatal suture differentiated into osteoblasts under the stimulation of expansion tension, while in the E + PTH + R group, there was more new bone formation (Fig. 4C, H). The results from TRAP staining indicated that in the control group, osteoclasts were distributed at the junction of the bony lamella and the connective tissue; in the PTH group, osteoclast was increased and cartilage matrix was locally absorbed; in the E group, osteoclasts extended toward the palate and bone resorption range was increased; in the E + PTH group, bone remodeling was markedly enhanced; in the E + R and E + PTH + R group, osteoclasts and bone resorption were evident at the margin of the bone (Fig. 4D, I).

**Fig. 5:**
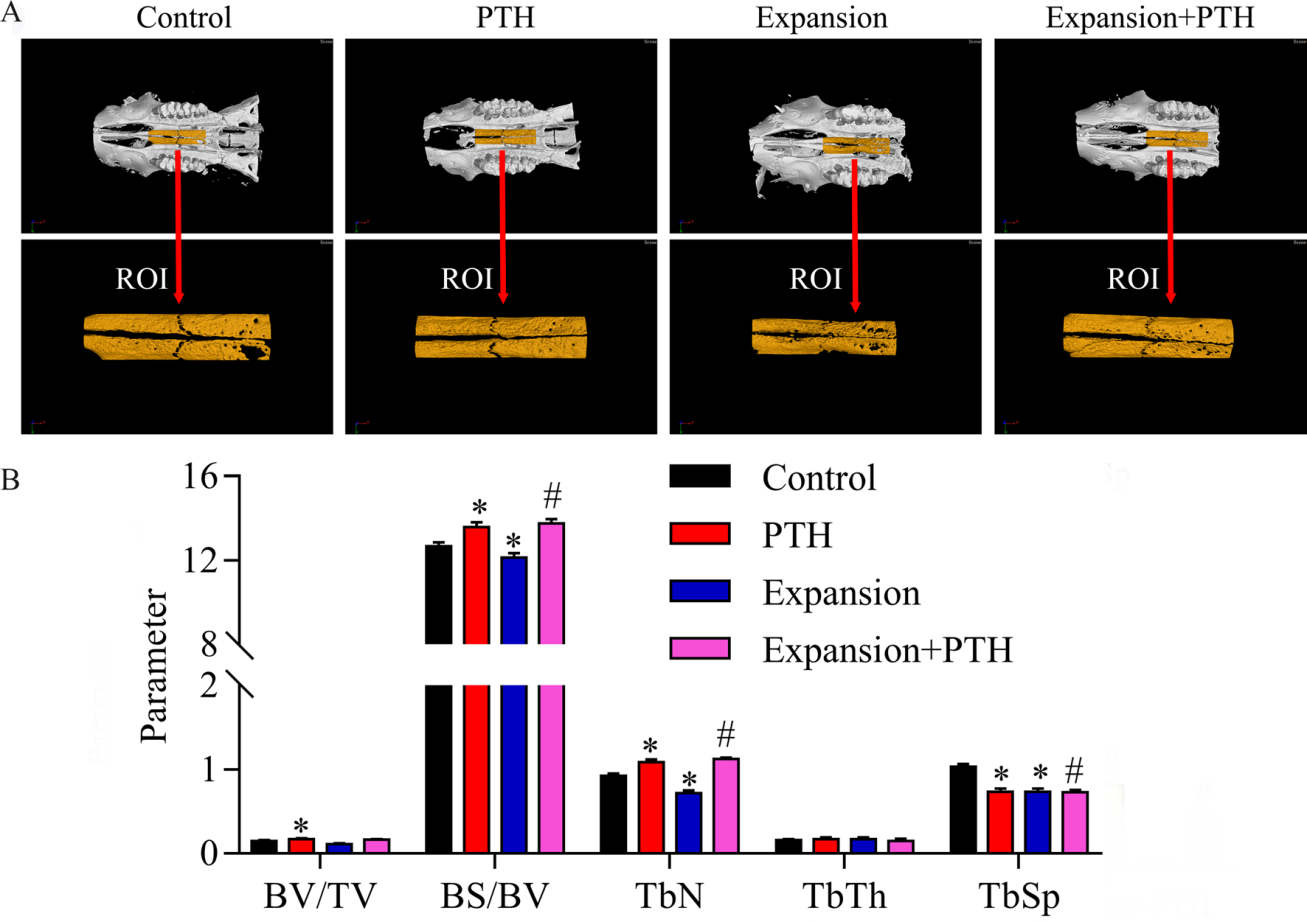
PTH accelerated maxillary reconstruction of maxillary expansion model rats. The rats were divided into control, PTH, expansion and expansion + PTH groups, respectively. **A** The bone rebuilding of maxillary expansion model rats was determined using microCT method. **B** The BV/TV, BS/BV, TbN, Tb.Th and Tb.Sp were quantized, respectively. *P < 0.05 versus control group; #*P* < 0.05 versus expansion group. BV/TV, trabecular bone volume fraction; BS/BV, smaller specific bone surface; TbN, trabecular number; Tb.Th, trabecular thickness; Tb.Sp, trabecular separation

According to the results of micro-CT analyses, we found that trabecular bone volume fraction (BV/TV), smaller specific bone surface (BS/BV) and trabecular number (TbN) were aggrandized, while trabecular separation (Tb.Sp) was decreased in the PTH group compared with the C group; BV/TV, BS/BV and TbN were reduced, while Tb.Sp was raised in the E group relative to the C group; BV/TV, BS/BV and TbN were increased, while Tb.Sp was decreased in the E + PTH group relative to the E group (Fig. 5).

### PTH up-regulated RANKL, β-catenin, collagen II and OCN expressions in the rat maxillary expansion model

**Fig. 6:**
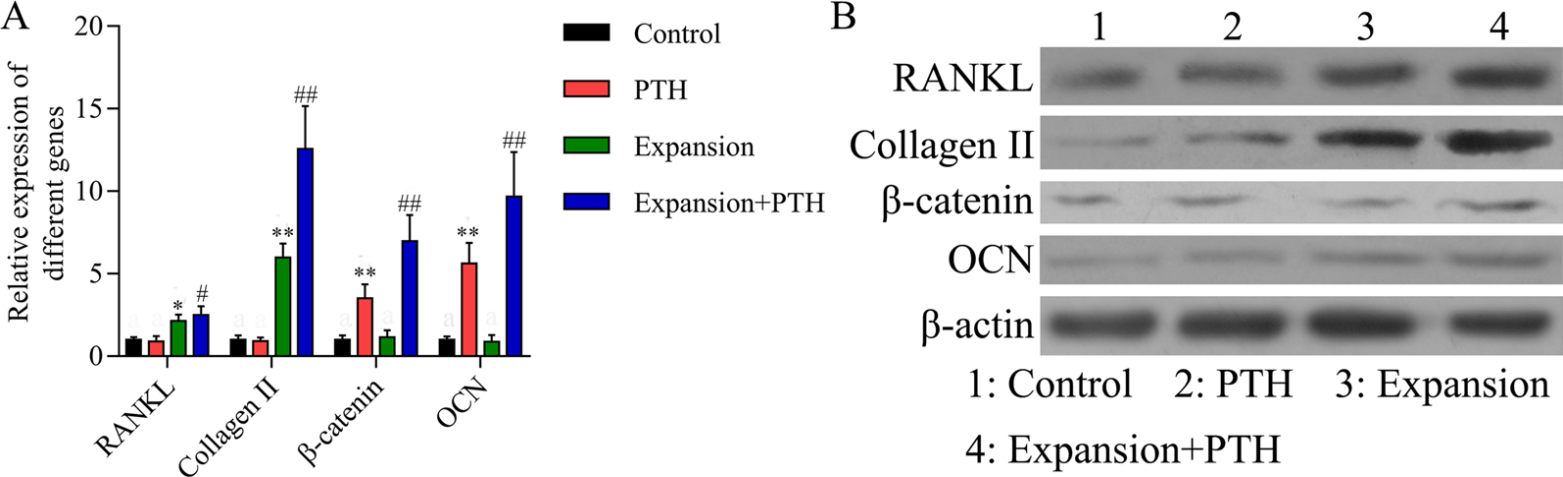
PTH up-regulated RANKL, β-catenin, collagen II and OCN expressions in maxillary expansion model rats. The rats were divided into control, PTH, expansion and expansion + PTH groups, respectively. **A** The mRNA expression levels of RANKL, β-catenin, collagen II and OCN were analyzed by RT-qPCR assay, *P < 0.05, **P < 0.01 versus control group; #*P* < 0.05, ##*P* < 0.01 versus expansion group. **B** RANKL, β-catenin, collagen II and OCN expressions were examined by western blot assay in each group. β-actin was used as internal reference. Different letters marked were considered to have significant difference between the two groups

In order to further explore the possible mechanisms of PTH on bone remodeling of maxillary expansion model rats, RT-qPCR and western blot assays were utilized to assess RANKL, β-catenin, collagen II and OCN expressions. As shown in Fig. 6, RANKL, β-catenin, collagen II and OCN expressions were up-regulated in the E and E + PTH groups relative to the C group; RANKL, β-catenin, collagen II and OCN expressions were up-regulated in the E + PTH group compared with the PTH or E group. Therefore, we suggested that PTH promoted the formation of cartilage and new bone in the mid-palatal suture through the up-regulation of the Wnt/β-catenin and RANKL pathways.

## Discussion

For many years, scholars have tried to find an effective and convenient way to prevent the relapse of mid-palatal suture expansion [28, 29]. There is no doubt that mechanical retention is the most effective way to achieve this goal. However, most patients will undergo a long retention period after expansion, which inevitably gives rise to many problems, such as poor oral hygiene, occlusal interference, and maxillofacial deformities [30]. Therefore, more advanced and effective approaches are called for to shorten the mechanical retention time after operation and reduce relapse.

Rats and mice are commonly used for maxillary expansion experiments [31, 32], in which their palate structure and sutural responses have been well investigated. In contrast to humans, whose mid-palatal suture features a fibrous interdigitated interface since 3 years old, the rats and mice have a straight synchondrotic suture mainly consisting of secondary cartilage even in their adulthood [33, 34]. Despite different characteristics of mid-palatal sutures, humans and mice share anatomical similarity in the structure of osseous palates. Their palatal bones are joined by sutures in anatomical planes transversely and sagittally, which allows the palate to grow in two directions [35]. While stimulated by expansion force, the mid-palatal suture functions as a growth site for increasing maxillary width [36]. Prior studies have suggested that rats are the ideal animals to obtain bone and suture changes under tension, and the individual differences of rats were small in the same age-group, which could minimize the experimental errors [37]. In our study, we selected 6-week-old rats, whose development stage was equivalent to human of 12 years old [38]. This age was just the appropriate stage for orthodontic treatment and arch expansion in clinical practice. Maxillary expansion in rats can be divided into maxillary anterior region [39] and maxillary posterior tooth area [40, 41]. Since the structures of molar, maxilla and mid-palatal suture in the posterior tooth area are more similar to those in humans, and the basic principles of mechanical tension in distraction osteogenesis are similar to those in clinical practice [42], we conducted maxillary expansion in the posterior area.

The expansion device in the maxillary posterior teeth of rats mainly includes a “U”- or “W”-shaped spring [14]. Generally, the free arms of spring are bonded to the occlusal or palatal sides of the molars, or the end of the free arm is directly inserted under the adjacent point of the molars to fix the arch spring. After the arms are bonded and fixed, the spring cannot be easily reactivated. In the present study, we adopted a “W”-shaped spring in combination with palatal tubes, which can easily be taken out for reactivation. We found that the RA expansion device significantly increased the maxillary arch widths, more than the conventional SA device did. Moreover, the operation difficulty of the repeated activation devices was reduced by respective bonding of bilateral palatal tubes, with larger diameter compared to the traditional spring (0.6 mm for the former, 0.35 mm for the latter), which increased the bonding area and may reduce the shedding rate. Thus, this novel rat maxillary expansion model can be suggested in future studies, especially for observation of prolonged expansion.

Physiologically, there are two main types of bone formation during mid-palatal suture expansion: intramembranous osteogenesis and endochondral osteogenesis [43]. Histological study showed that mesenchymal progenitor cells were in the center of palatal suture, mature hypertrophic chondrocytes were on both sides, maxillary plate with bone marrow cavity was in the lateral cartilage, and a small number of osteoblasts were in the bone margin [9], which were proved in this study. Mesenchymal precursors have great potential for proliferation and differentiation, and can respond to different mechanical forces [44]. Studies have indicated that compressive force could accelerate the differentiation of progenitor cells into chondrocytes [45], and tension force could induce the differentiation into osteoblasts [11]. In addition, in the process of maxillary expansion, there were both bone resorption and bone formation in the mid-palatal suture, which was called bone remodeling [9]. Obviously, the rate of bone remodeling decided the expansion width and speed, which was controlled by the device-applied tension. As our results showed, the mid-palatal suture was dilated, mesenchymal precursors proliferated, cartilages increased, and there were osteoclasts and bone resorption area at the periosteal margin in the SA and RA groups.

Relapse is always inevitable although significantly improved mid-palatal suture width is acquired after expansion and a long maintenance period. A recent systematic review reported that at least 10% post-retention relapse occurred [46]. The reason is that a malformed balance with the dental arch, tongue and buccal muscles already exists before expansion and the newly formed mid-palatal sutures subjected to compressive force from the buccal-inclined upper posterior tooth, the lingual-inclined lower posterior tooth, the relatively small tongue or the nervous buccal muscles. Moreover, the new mid-palatal suture cannot form enough bone to resist the compression force, leading to the relapse [47]. In conclusion, enhancing bone remodeling and new bone formation via external interventions in palatal suture during expansion and retention is considered to be the key to reduce relapse.

PTH is one of the most important peptide hormones regulating calcium and phosphorus metabolism and bone conversion, which is currently a promising bone formation promoter [48]. Considerable researches have supported the regulatory effect of PTH on bone remodeling [49, 50]. In the present study, we found that PTH markedly increased osteoblasts, osteoclasts and chondrocytes number in the mid-palatal suture during expansion, suggesting that PTH improved bone remodeling of the expansion. The Col-I expression staining indicated that PTH promoted the bone formation, which was further supported by the micro-CT results that BV/TV, BS/BV and TbN were all increased. The TRAP staining indicated that osteoclasts and bone resorption were pronounced at the margin of the bone. These results are consistent with previous studies that continuous PTH administration can both stimulate bone formation and bone resorption [51]. On the one side, RANKL is a bone resorption promoting factor produced by osteoblasts and progenitors, which could activate the specific receptor (RANK) on the cell membrane of osteoclast progenitors, induce the formation and activation of osteoclasts, and participate in the regulation of bone resorption [52]. In the process of expansion, PTH could bind to the PTHR1 receptor of mid-palatal suture, leading to increased RANKL expression, which promoted bone resorption and bone remodeling [53]. On the other side, the enhanced bone formation by PTH in this study was attributed to its anabolic effect, which was proved by the increasing expression of collagen II and OCN, which are markers of cartilage and bone formation, respectively [54, 55]. This effect was regulated by osteoclast-dependent and osteoclast-independent (Wnt signaling) pathways [56]. Hence, other than the up-regulated RANKL expression, we also investigated the Wnt/β-catenin pathway. β-catenin is a classical Wnt transmitter that is involved in regulating the proliferation of precursors and the maturation of osteoblasts during mid-palatal suture distraction osteogenesis [57]. In this study, the β-catenin expression was up-regulated by PTH and expansion, indicating that both the osteoclast-dependent and osteoclast-independent pathways took part in the mechanisms of the stimulation effect on bone remodeling during expansion.

Nevertheless, the authors have to admit that there were some limitations in the present study. The sample number in the relapses groups was small, and a group with PTH administration starting at retention may be even better to explore the actual effect of PTH on retention with a longer retention time. Thus, the roles of PTH administration in the treatment of maxillary constriction, which is interesting and promising according to our findings, need further studies in future.

## Conclusion

The novel repeated activation maxillary expansion device had better mid-palatal expansion effects than the conventional single activation device and therefore could be suggested in future studies. PTH further promoted bone remodeling and reduced relapse in rat maxillary expansion, partially by regulating Wnt/β-catenin and RANKL pathways.

## Data Availability

The datasets used and/or analyzed during the current study are available from the corresponding author on reasonable request.

## Abbreviations

PTH: Parathyroid hormone
SA: Single activation
microCT: Micro-computed tomography
BV/TV: Bone volume/total volume
BS/BV: Smaller specific bone surface
TbN: Trabecular number
Tb.Th: Trabecular thickness
Tb.Sp: Trabecular separation
GAPDH: Glyceraldehyde 3-phosphate dehydrogenase
